# Brain Metastases in Cervical Cancer: A Global Systematic Review and Meta‐Analysis of Incidence and Clinicopathological Features

**DOI:** 10.1002/cnr2.70405

**Published:** 2025-11-26

**Authors:** Kimia Pakdaman, Amirhossein Alizadeh‐Nodehi, Amir‐Hossein Lashkarbolouki, Amin Esmaeilnia Shirvani, Kasra Pakdaman, Saba Sahraian, Hossein‐Ali Nikbakht, Mosua Yeminfroz, Pouyan Ebrahimi

**Affiliations:** ^1^ School of Medicine Babol University of Medical Sciences Babol Iran; ^2^ Student Research Committee Babol University of Medical Sciences Babol Iran; ^3^ School of Nursing and Midwifery Mazandaran University of Medical Sciences Sari Iran; ^4^ Department of Neural Science, College of Arts and Sciences New York University New York New York USA; ^5^ Social Determinants of Health Research Center, Health Research Institute Babol University of Medical Sciences Babol Iran; ^6^ Cancer Research Center, Health Research Institute Babol University of Medical Sciences Babol Iran

**Keywords:** brain neoplasm, cervical neoplasm, incidence, meta‐analysis, metastases

## Abstract

**Background:**

Cervical cancer (CC) remains the fourth most prevalent malignancy among women globally, with a disproportionate burden in low‐ and middle‐income countries, where it is often diagnosed at advanced or metastatic stages.

**Aims:**

Despite an increasing number of case reports and institutional studies on brain metastases (BMs) arising from CC, current understanding of their epidemiology, clinical presentation, and prognostic implications remains fragmented and lacks comprehensive synthesis.

**Methods:**

We executed a systematic and unrestricted search of five major databases, PubMed/Medline, Scopus, Embase, Web of Science, and Google Scholar, covering all records up to April 19, 2025. Studies were eligible for inclusion if they provided a clear report on the incidence of BMs among CC patients. The quality and risk of bias of the selected studies were independently appraised using the Joanna Briggs Institute (JBI) assessment criteria. For statistical analysis, we utilized STATA version 17, implementing random‐effects meta‐analytical models to estimate the aggregated incidence along with corresponding 95% confidence intervals (CIs).

**Results:**

A total of 17 studies encompassing 33 datasets were included in the final analysis. The global pooled incidence of BMs among cervical cancer patients was estimated at 0.65% (95% CI: 0.46–0.85). Incidence was highest in Turkey (1.83%, 95% CI: 0.91–2.75) and lowest in South Africa (0.22%, 95% CI: 0.0–0.47). Among histologic subtypes, neuroendocrine carcinoma exhibited the highest pooled incidence of BMs at 10.60% (95% CI: 0.0–21.63), followed by adenocarcinoma at 0.89% (95% CI: 0.14–1.64). The pooled mean survival time following the diagnosis of BMs was 6.80 months (95% CI: 5.08–8.52), while the mean interval from the initial cervical cancer diagnosis to the development of BMs was 28.15 months (95% CI: 24.27–32.03).

**Conclusion:**

Although BMs in cervical cancer are rare, they are associated with dismal survival outcomes and poor prognosis. These findings underscore the importance of vigilant surveillance in high‐risk patients and may inform the development of more targeted and effective therapeutic and preventive strategies.

## 1 Introduction

Globally, Cervical cancer (CC) ranks as the fourth most prevalent malignancy among women worldwide, following breast, lung, and colorectal cancers. Each year, it accounts for approximately 661 000 new cases and over 348 000 deaths globally. Notably, it holds the highest incidence among gynecologic malignancies in 25 countries and is the leading cause of cancer‐related mortality among women in 37 nations [1, 2]. The global burden of CC is disproportionately concentrated in low‐ and middle‐income countries (LMICs), where the incidence and mortality rates are two‐ and five‐fold higher, respectively, compared to high‐income countries (HICs) [3]. This disparity is largely attributed to limited access to organized cervical cancer screening programs, inadequate healthcare infrastructure, and low uptake of preventive services such as HPV vaccination, leading to delayed diagnosis and treatment [4, 5]. Also, in these settings, diagnosis is frequently delayed, with 75%–90% of cases identified at advanced clinical stages, when the disease has often already invaded adjacent structures or metastasized distantly, rendering standard therapeutic interventions largely ineffective [6, 7].

Metastatic dissemination occurs in approximately 25% of CC cases [8]. Unlike patients with localized or regionally advanced disease, for whom standard treatment protocols exist, there is currently no universally established therapy for those presenting with distant metastases [9, 10, 11]. As a result, these patients face significantly poorer outcomes, with a five‐year survival rate of only 17%, compared to much higher rates among those with localized disease [12]. Although lymphatic spread to pelvic and para‐aortic lymph nodes is the most common route of dissemination, hematogenous spread also plays a critical role. The liver, lungs, and bones are the most frequent sites of distant metastases, with reported incidence rates varying across studies: according to Li et al., the incidence of brain metastases is 0.46%, compared to 2.29% for liver, 3.86% for lungs, and 2.10% for bones [13]; similarly, Park et al. reported incidences of 3.17% for lungs, 1.54% for liver, and 1.97% for bones [14], highlighting that brain metastases are markedly less frequent than other distant sites. Importantly, the metastatic pattern varies according to the histologic subtype of CC. Pulmonary and osseous metastases are more common in squamous cell carcinoma, whereas neuroendocrine carcinoma (NEC) typically spreads to the liver and lungs. Moreover, patients with neuroendocrine histology demonstrate a markedly higher rate of multiorgan metastases, observed in approximately 44.4% of cases [15]. Brain metastases (BMs) in CC are rare, with a reported incidence ranging between 0.2% and 2% [14, 16]. Nevertheless, an upward trend in the number of reported cases has been noted over the past two decades, likely attributable to advances in imaging techniques and extended survival in patients with advanced disease [17].

Given the rarity of BMs originating from CC and the limited number of available studies [18, 19], essential characteristics, including incidence rates, clinicopathological features (e.g., presenting symptoms, concurrent metastatic burden, and histology‐specific BMs rates), and survival outcomes, remain inadequately characterized. In this context, a recent systematic review by Kato et al. in 2021 provided a broader overview of incidence, clinical presentation, treatment strategies, survival, and prognostic factors among patients with cervical and endometrial cancer [17]. Despite its breadth, that review did not include a meta‐analysis, thus limiting the interpretability and generalizability of its findings. In response, we aimed to conduct a comprehensive systematic review and meta‐analysis to better quantify the incidence, characteristics, and outcomes of BMs in CC patients.

## 2 Materials and Methods

### 2.1 Study Design

Following the principles outlined in the PRISMA statement, we designed and reported this study to ensure transparency and methodological rigor (PROSPERO registration number: CRD420251068706) [20].

### 2.2 Search Strategy and Data Sources

We developed a multi‐step search protocol to systematically identify relevant studies. Comprehensive database searches were conducted in PubMed/Medline, Scopus, Embase, Web of Science, and Google Scholar without applying any filters or restrictions, covering all available literature up to April 19, 2025. Additionally, gray literature sources were explored to minimize the risk of publication bias and ensure a more exhaustive evidence base. The search strategy was constructed using a combination of Medical Subject Headings (MeSH) and free‐text keywords relevant to the field. Terms were derived from the PubMed MeSH thesaurus and supplemented with domain‐specific vocabulary to enhance sensitivity. Core search terms included: “Brain Neoplasm”, “Brain”, “Metastases”, “Cervical Neoplasm”, and “Incidence” (Table S1).

### 2.3 Study Selection Process

All identified records were compiled and managed using EndNote 2019 (version X9; Clarivate, Philadelphia, PA, USA). Duplicate entries were detected and excluded using the software's built‐in duplication detection tool. Two independent reviewers then screened the remaining articles to determine their suitability for inclusion. Any disagreements in judgment were addressed by a third investigator, who served as the lead researcher for the study.

Eligibility was limited to studies that explicitly reported the frequency of BMs in CC patients, including both the total sample size (non‐metastatic CC patients) and the number of affected cases. Only articles that offered clear and quantifiable incidence data were retained for analysis. Exclusion criteria were applied to filter out the following: (1) case reports, case series, experimental studies (in vivo or in vitro), book chapters, conference abstracts, and literature reviews; (2) studies focusing solely on advanced‐stage CC (FIGO stage IVb or M1); (3) investigations using duplicate or overlapping databases; and (4) papers that reported BMs from gynecologic malignancies without specifying CC as the primary site. A substantial portion of potentially eligible studies originated from the SEER (Surveillance, Epidemiology, and End Results) registry [21]. From this pool, only those that offered the most comprehensive incidence data, based on rigorous inclusion/exclusion standards and well‐defined study periods, were selected for inclusion. In cases where full‐text access was not readily available, the corresponding authors were contacted by email. Studies were excluded if no response or article access was obtained.

### 2.4 Data Extraction and Variables

Following the acquisition of full‐text PDFs for all eligible articles, two researchers independently conducted data extraction using Microsoft Excel 2019 (Microsoft Corp., Redmond, WA, USA). Any discrepancies during the extraction process were reviewed and resolved by the principal investigator overseeing the study. The data extraction template was predefined and included comprehensive study‐level and patient‐level variables: name of the first author, publication year, time span of data collection, patient age range, study design, geographic classification based on WHO regions, country of origin, Human Development Index (HDI) ranking, income level, FIGO stage of CC, tumor histology, and the count of BMs categorized as single, multiple, or total. Additionally, a combination approach was applied to ensure thorough data capture: during extraction, we also examined important additional variables not included in the original template, including clinical presentations (e.g., headache, weakness, nausea/vomiting, paralysis, seizures, confusion), number of metastatic sites in the brain (solitary vs. multiple), patient survival outcomes, and reported incidence rates. This hybrid approach allowed us to extract all relevant information from each study, ensuring a comprehensive and high‐quality dataset for our meta‐analysis. Countries were mapped to one of the six WHO‐designated regions (Africa, Eastern Mediterranean, Europe, South‐East Asia, the Americas, Western Pacific) [22], and stratified by HDI status [23]. Tumor staging was classified according to the FIGO system (Stages I–IV) [24], while histological types were divided into three main categories: adenocarcinoma (ADC), squamous cell carcinoma (SCC), and NEC [16]. Classification of metastases as single or multiple was determined either by the presence of extracranial metastases or the original study's definition. Clinical manifestations were grouped into six predefined symptom categories. Survival outcomes were measured using two endpoints: (1) survival time (from diagnosis of BMs to death), and (2) disease interval (from initial CC diagnosis to onset of BMs) [25].

### 2.5 Quality Appraisal (Risk of Bias Assessment)

The quality of the studies included in this review was appraised using the Joanna Briggs Institute (JBI) “Checklist for Prevalence Studies.” Two reviewers independently applied the checklist to evaluate each study. Any disagreements that arose during this process were resolved by the principal investigator. Studies were scored accordingly: those earning 5–6 points were rated as having moderate quality; scores greater than 6 indicated high quality (low risk of bias), and scores below 5 reflected low methodological quality (high risk of bias) [26, 27].

### 2.6 Statistical Analysis

All statistical analyses were conducted using STATA software, version 17 (StataCorp, College Station, TX, USA). To estimate the pooled incidence of BMs among patients with CC, a random‐effects model method was employed. This approach accounts for potential clinical and methodological heterogeneity across included studies. Effect sizes were calculated as raw (untransformed) proportions, accompanied by 95% confidence intervals (CIs). Statistical heterogeneity was assessed using the *I* ^2^ statistic, with *I* ^2^ values of 25%, 50%, and 75% interpreted as low, moderate, and high heterogeneity [28, 29, 30, 31]. A forest plot was generated to visually represent pooled incidence rates along with inter‐study variability. To explore potential sources of heterogeneity, subgroup analyses were conducted based on publication year, data collection period, country of origin, income level, risk of bias classification, and tumor histology. Sensitivity analysis using a leave‐one‐out approach was performed to assess the robustness of the overall pooled estimate. Additionally, meta‐regression was applied to evaluate the impact of continuous variables, such as publication year, HDI level, mean age of patients with BMs, and start year of data collection. Descriptive analyses were also performed to summarize key clinical characteristics in patients with BMs, including symptom profiles, number of brain lesions, CC stage, and the presence or absence of extracranial metastases. Furthermore, we used a meta‐analysis of continuous outcomes to estimate the pooled mean survival time and diagnostic interval (from CC diagnosis to BMs) in this population [32, 33]. For studies reporting descriptive statistics as medians with ranges or interquartile ranges (IQRs), or presenting individual case‐level data, mean and standard deviation (SD) estimates were derived using the Meta‐Analysis Accelerator platform [34]. Potential publication bias was examined both visually using a funnel plot and analytically via Egger's regression test. A *p*‐value < 0.05 was considered indicative of asymmetry and potential publication bias [29]. All analyses were performed in accordance with established guidelines for meta‐analyses of prevalence data, ensuring transparency, reproducibility, and methodological rigor [35].

## 3 Results

### 3.1 Study Selection

**FIGURE 1:**
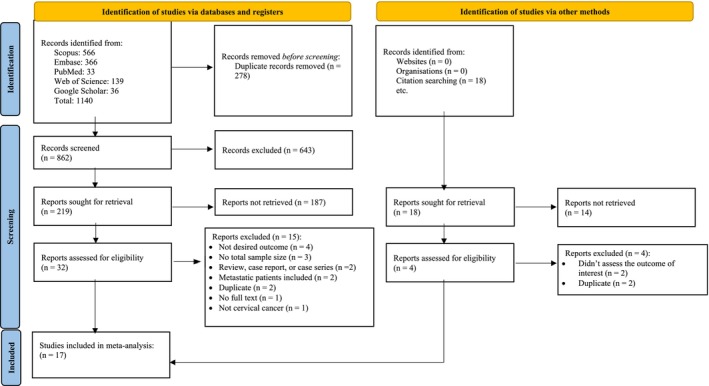
PRISMA flow diagram.

Based on the predefined search strategy, a total of 1140 articles were initially retrieved for screening. After removing 278 duplicates, 862 unique records were included in the title and abstract screening phase. Following the application of eligibility criteria, 32 articles proceeded to full‐text review. Ultimately, 17 studies met the inclusion criteria and were deemed eligible for the final meta‐analysis. These studies collectively contributed 33 data records, which were compiled and analyzed using Microsoft Excel for quantitative synthesis (Figure 1).

### 3.2 Study Characteristics and Quality Appraisal

Among the 17 included studies, the publication years ranged from 1985 to 2024, while the data collection periods spanned from 1972 to 2014. All studies employed a retrospective design and drew their data from 10 distinct countries. Specifically, one record was contributed by each of the following countries: Italy, China, Brazil, Turkey, Mexico, Iran, and South Africa. South Korea, Japan, and the United States contributed two, three, and five records, respectively. Notably, the study by Park et al. [14] was counted as two separate records due to the use of two independent databases with distinct data collection periods. From a geographic perspective, the majority of records originated from the Region of the Americas (*n* = 8) and the Western Pacific Region (*n* = 6). According to the HDI and national income classifications, most studies were conducted in countries categorized as “high” or “very high” in terms of socioeconomic development. Only one study focused exclusively on patients with adenocarcinoma (ADC) histology [13], whereas the remaining studies included CC patients regardless of histologic subtype. Quality appraisal using the JBI Prevalence Studies Checklist revealed that 3 studies were rated as low quality, 7 as moderate quality, and 7 as high quality (Table 1).

**TABLE 1:** Basic information of included studies.

ID	Author (published year)	Start‐year of data collection	End‐year of data collection	Age range	Age mean (patients)	Age SD	Country	Histology of cervical cancer patients	Sample size of cervical cancer (number)	Patients with brain metastasis (number)	Risk of bias
1	Barmeir et al. (1985) [36]	1981	1985	NA	NA	NA	South Africa	All type	1347	3	High
2	Cacho‐Dı'az et al. (2016) [37]	2009	2015	33–70	50.00	11.00	Mexico	All type	2637	27	Moderate
3	Cagney et al. (2017) [38]	2010	2013	≥ 18	NA	NA	USA	All type	12 577	48	Low
4	Chura et al. (2007) [39]	1995	2006	29–57	42.50	9.48	USA	All type	1565	12	Low
5	Cormio et al. (1996) [40]	1982	1994	28–62	50.25	10.83	Italy	All type	1184	14	High
6	Nasioudis et al. (2020) [25]	2010	2015	NA	55.00	12.35	USA	All type	57 160	211	Moderate
7	Hwang et al. (2013) [41]	2001	2011	33–75	52.73	12.40	Republic of Korea	All type	2458	11	Low
8	Ikeda et al. (1998) [42]	1974	1994	41–73	53.75	12.39	Japan	All type	1961	8	Moderate
9	Li et al. (2024) [13]	2010	2019	≥ 18	NA	NA	USA	ADC	7201	33	Low
10	Park et al. (2022) [14]	1994	2014	NA	NA	NA	Republic of Korea	All type	3929	27	Moderate
		2010	2019	NA	NA	NA	USA	All type	26 959	93	
11	Rangel et al. (2022) [16]	2010	2017	≥ 18	NA	NA	Brazil	All type	3397	51	Low
12	Saphner et al. (1989) [43]	1972	1986	19–93	NA	NA	USA	All type	1219	6	High
13	Sun et al. (2020) [44]	2000	2019	≥ 18	48.90	10.30	China	All type	7098	24	Moderate
14	Tabatabaei et al. (2022) [45]	2014	2020	22–85	NA	NA	Iran	All type	187	1	Moderate
15	Takeshita et al. (2017) [46]	1995	2015	37–80	59.50	11.53	Japan	All type	1046	18	Low
16	Teke et al. (2015) [47]	1996	2006	32–77	50.00	12.96	Turkey	All type	820	15	Moderate
17	Kim et al. (2019) [48]	1995	2016	NA	54.11	12.29	Republic of Korea	All type	2774	19	Low

*Note:* ADC, adenocarcinoma; NA, not available; SD, standard deviation; USA, United States of America.

### 3.3 Results of the Meta‐Analysis

#### 3.3.1 Brain Metastases Cervical Cancer Global Incidence

Table 2 presents the pooled incidence of BMs among CC patients, both globally and stratified by country. Based on 18 data records extracted from the included studies, comprising a total of 135 519 patients worldwide, the overall pooled incidence of BMs was estimated at 0.65% (95% CI: 0.47–0.83), with substantial heterogeneity observed across studies (*Q* = 82.74, *I* ^2^ = 94.49%), reflecting considerable variability in study design, populations, and reporting methods. Among individual countries, Turkey reported the highest incidence, with a single study yielding a rate of 1.83% (95% CI: 0.91–2.75). Brazil and Italy followed, each with one contributing record and incidence rates of 1.50% (95% CI: 1.09–1.91) and 1.18% (95% CI: 0.57–1.80), respectively. In contrast, South Africa recorded the lowest incidence, at 0.22% (95% CI: 0.00–0.47), based on one study. China and the United States also reported relatively low pooled incidence rates: 0.34% (95% CI: 0.20–0.47) from a single Chinese study and 0.37% (95% CI: 0.34–0.41) across six U.S. records (Table 2).

**TABLE 2:** Pooled incidence of brain metastases in cervical cancer based on countries.

Country^a^	Number of datasets	Number of cervical cancer patients (total)	Number of patients with brain metastasis	Pooled incidence per 100 (95% CI)	Heterogeneity	
					*Q*	*I* ^2^ (%)
USA	6	106 681	403	0.37 (0.34–0.41)	5.35	0.0
Korea	3	9161	57	0.60 (0.44–0.76)	2.01	5.60
Japan	2	3007	26	1.01 (0.0–2.29)	9.45	89.42
China	1	7098	24	0.34 (0.20–0.47)	—	—
Brazil	1	3397	51	1.50 (1.09–1.91)	—	—
Italy	1	1184	14	1.18 (0.57–1.80)	—	—
Turkey	1	820	15	1.83 (0.91–2.75)	—	—
Mexico	1	2637	27	1.02 (0.64–1.41)		
South Africa	1	1347	3	0.22 (0.0–0.47)	—	—
Iran	1	187	1	0.53 (0.0–1.58)	—	—
Total pooled incidence	18	135 519	621	0.65 (0.47–0.83)	82.74	94.49

^a^ Countries are sorted based on the number of datasets.

### 3.4 Subgroup Meta‐Analysis of Brain Metastasis Incidence Based on Priori Variables

Subgroup analyses based on pre‐specified variables are summarized in Table 3. The pooled incidence of BMs among CC patients was 0.73% (95% CI: 0.46–1.00) in studies published after 2015. Additionally, studies in which data collection began before the year 2000 reported a higher pooled incidence of 0.76% (95% CI: 0.47–1.05). With regard to study quality, a notable difference was observed. Studies classified as having a high risk of bias demonstrated a pooled incidence of 0.57% (95% CI: 0.05–1.09), whereas studies with low risk of bias yielded a higher pooled incidence of 0.78% (95% CI: 0.43–1.13). Income‐level stratification revealed that countries in the upper‐middle income category reported the highest pooled incidence at 0.86% (95% CI: 0.35–1.38), while studies from HICs reported a lower incidence of 0.50% (95% CI: 0.39–0.60) (Table 3).

**TABLE 3:** Incidence estimates for Brain metastases cervical cancer, according to a priori‐defined subgroups.

Variable subgroup	Number of datasets	Number of cervical cancer patients (total)	Number of brain metastasis	Pooled incidence per 100 (95% CI)	Heterogeneity	
					*Q*	*I* ^2^ (%)
Publish year						
Before 2015	6	9734	54	0.50 (0.30–0.70)	10.76	51.01
2015–2025	12	125 785	567	0.73 (0.46–1.00)	71.46	97.26
Start sampling date						
Before 2000	9	15 845	122	0.76 (0.47–1.05)	30.04	80.52
2000–2025	9	119 674	499	0.57 (0.32–0.81)	42.48	96.86
Risk of bias						
High	3	3750	23	0.57 (0.05–1.09)	8.32	80.06
Moderate	8	100 751	406	0.56 (0.34–0.77)	27.41	93.80
Low	7	31 018	192	0.78 (0.43–1.13)	40.13	92.86
Income level						
Upper‐middle	6	15 486	121	0.86 (0.35–1.38)	48.23	92.31
High	12	120 033	500	0.50 (0.39–0.60)	32.35	75.31

### 3.5 Co‐Occurrence of Brain Metastases and Associated Clinical Patterns

The analysis of co‐occurring metastases revealed that BMs were accompanied by metastases to other organs in approximately 69.24% of cases (95% CI: 58.29–80.20). Among these, pulmonary metastases were the most frequently observed, reported in 44.10% of cases (95% CI: 30.83–57.37). Regarding CC staging, the majority of patients with BMs were initially diagnosed at stage II, accounting for 35.53% (95% CI: 23.29–47.77) of cases. Anatomical distribution of intracranial lesions was also assessed. The presence of multiple BMs was more common (54.56%; 95% CI: 46.50–62.62) than solitary lesions (36.44%; 95% CI: 28.11–44.76). From a symptomatologic perspective, headache was the most frequently reported neurological symptom (46.58%; 95% CI: 35.58–57.59), followed by generalized weakness (35.80%; 95% CI: 16.23–55.37) (Table S2).

**FIGURE 2:**
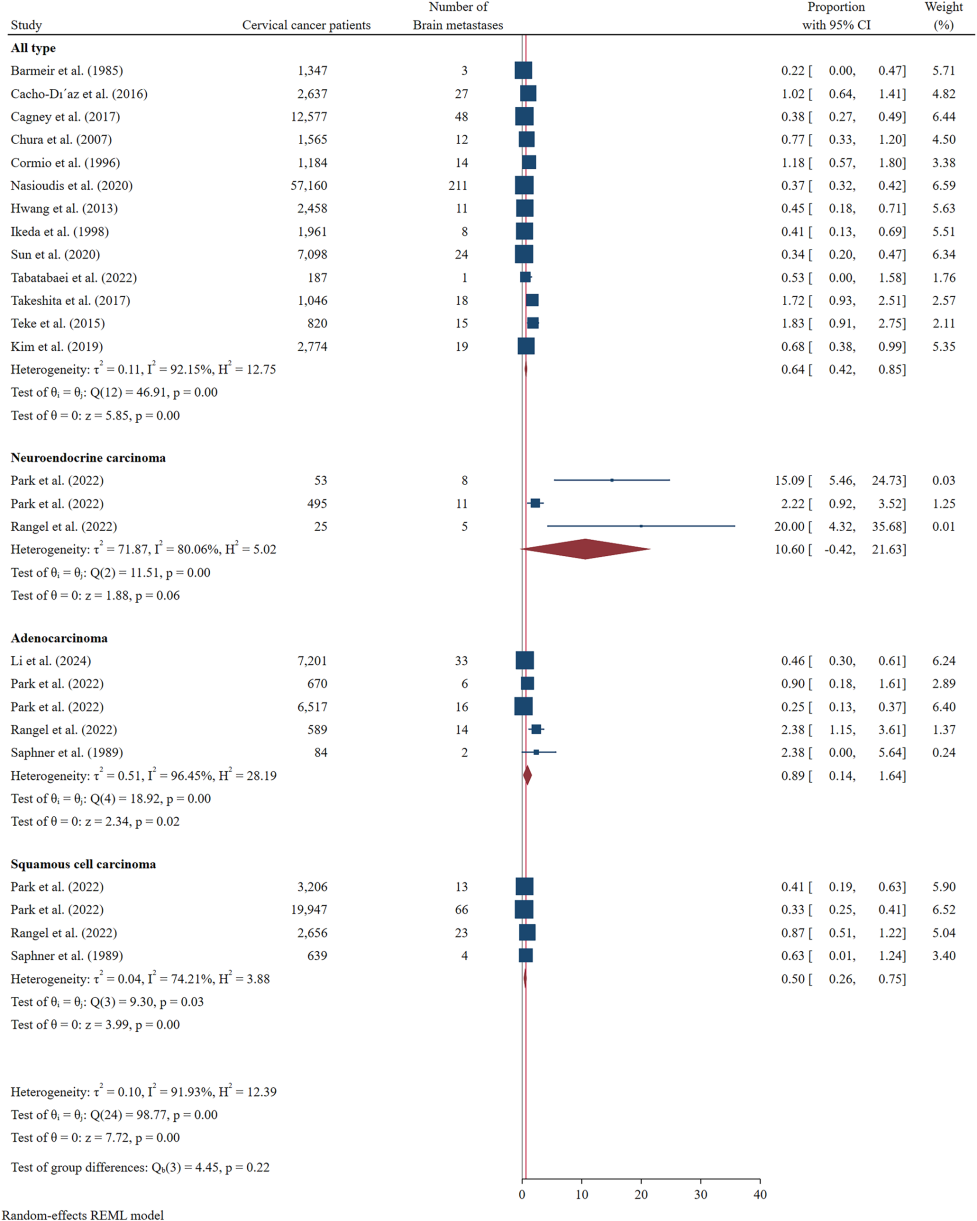
Forest plot of pooled incidence of brain metastases based on cervical cancer histology.

In histology‐based subgroup analyses, NEC exhibited the highest incidence of BMs at 10.60% (95% CI: 0.00–21.63), compared to ADC (0.89%; 95% CI: 0.14–1.64) and SCC (0.50%; 95% CI: 0.26–0.75) (Figure 2).

### 3.6 Meta‐Analysis of Continuous Outcomes (Survival and Interval)

**FIGURE 3:**
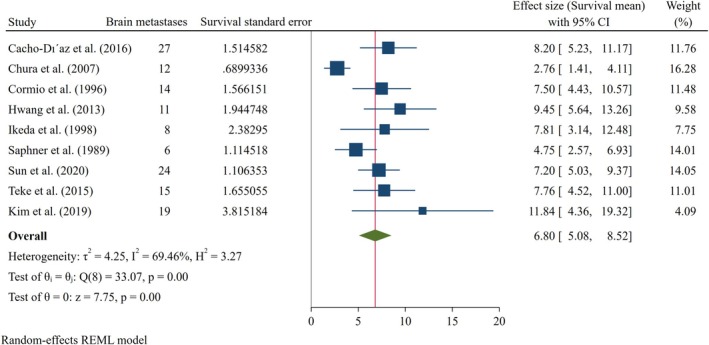
Forest plot of mean survival time of brain metastases patients.

**FIGURE 4:**
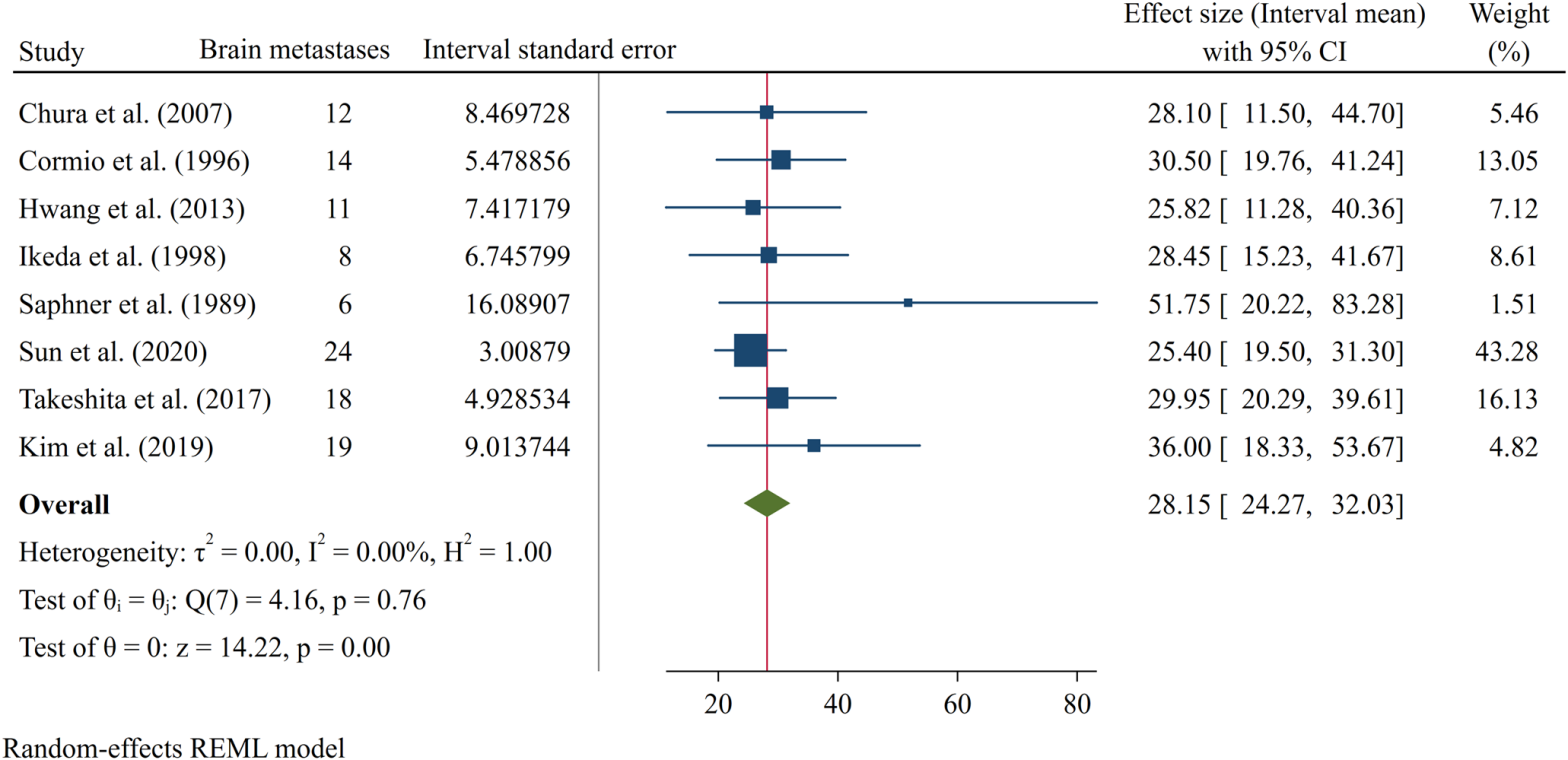
Forest plot of mean of interval time of cervical cancer patients till the occurrence of brain metastasis.

Pooled analysis of continuous outcomes demonstrated that the overall survival following the diagnosis of BMs among patients with CC was 6.80 months (95% CI: 5.08–8.52), as illustrated in Figure 3. Furthermore, the mean diagnostic interval, from initial CC diagnosis to the development of BMs, was estimated at 28.15 months (95% CI: 24.27–32.03) (Figure 4).

### 3.7 Meta‐Regression of Incidence of Brain Metastases

**FIGURE 5:**
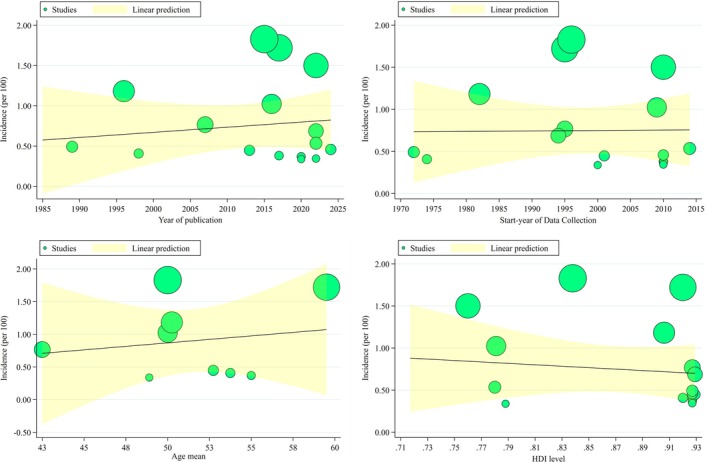
Meta regression of incidence of brain metastasis based on year of publication, start year of data collection, age mean, and HDI value.

Meta‐regression analyses were conducted to evaluate temporal and demographic trends in the pooled incidence of BMs. As illustrated in Figure 5, there was a non‐significant upward trend in incidence rates over time based on the year of publication (1985–2025), with a regression coefficient of 0.0052 (95% CI: −0.0110 to 0.0215; *p* = 0.526). Similarly, no significant change was observed in pooled incidence with respect to the year of data collection (1970–2015), yielding a regression coefficient of 0.0015 (95% CI: −0.0137 to 0.0168; *p* = 0.842). When stratified by mean patient age, the pooled incidence showed a non‐significant increase across studies with a higher average age, from 42.5 to 60 years (Coefficient = 0.0120; 95% CI: −0.0693 to 0.0933; *p* = 0.772). Additionally, an increase in the HDI was not significantly associated with changes in BMs incidence (Coefficient = −0.9028; 95% CI: −3.5779 to 1.7723; *p* = 0.508) (Figure 5).

### 3.8 Sensitivity Analysis and Publication Bias

Sensitivity analysis confirmed the robustness of results, as omitting individual studies did not significantly alter the pooled incidence of BMs (Figure S1). Visual inspection of the funnel plot, along with Egger's regression test (bias = 2.72; SE = 0.506; *p* < 0.001), revealed significant asymmetry (Figure S2).

## 4 Discussion

This systematic review and meta‐analysis, encompassing data from 132 745 CC patients, estimated a pooled incidence of BMs at 0.65 per 100 individuals. Notably, over two‐thirds of BMs co‐occurred with extracranial metastatic sites, most commonly the lungs (44.10%) and bones (22.97%). These findings align with previous large‐scale database analyses. For instance, Shan et al. [49], analyzing SEER data from 2010 to 2014, reported that the incidence of brain metastases ranged from 0.1% to 0.3% over the study years. In the same cohort, pulmonary metastases were the most frequent, occurring in 3%–4% of cases, whereas liver and bone metastases ranged between 1% and 3%, highlighting that brain metastases consistently remain the rarest distant site regardless of the year of study. Similarly, Wu et al. analyzed data from 25 476 CC patients between 2010 and 2019 and found 88 cases of brain metastases (0.35%). Importantly, they observed that co‐occurring metastases to the lungs, liver, or bones did not independently predict brain metastases, underscoring the relative rarity of brain involvement in CC [50]. These findings are broadly consistent with our pooled results, which estimated a BM incidence of 0.65% and demonstrated that the majority of BMs co‐occur with extracranial metastases, particularly the lungs and bones. It should be noted that substantial heterogeneity was observed across studies contributing to this pooled estimate (*I* ^2^ = 94.49%), and thus these findings should be interpreted cautiously. Overall, this comparison confirms that brain metastases in CC are rare and occur predominantly alongside other metastatic sites. A majority of the studies included in our meta‐analysis originated from HICs. In these settings, greater public awareness and broader access to screening programs have contributed to earlier detection of CC, often at non‐metastatic stages. Consequently, the probability of disease progression to distant metastasis, including brain involvement, is lower in these populations [51]. In contrast, in many LMICs, CC is typically diagnosed at more advanced stages due to limited screening infrastructure and lower healthcare accessibility. Moreover, a lack of robust cancer registries in these regions results in significant underreporting, thereby limiting the availability of population‐level data from LMICs [52, 53]. These observations reflect considerable geographic variability in reported BMs rates among CC populations. This imbalance may have introduced a selection bias in our analysis, skewing the overall findings toward data from more developed healthcare systems. Nevertheless, studies conducted in Brazil, Turkey, and Mexico reported relatively higher rates of BMs compared to high‐income settings, although such events remain exceedingly rare even in those countries [16, 37, 47]. It is also important to note that BMs from CC are rarely isolated. Most cases occur as part of widespread systemic dissemination, often involving the lungs, liver, and bones. Hematogenous spread is considered the primary route, and one proposed pathway involves tumor cell migration from pelvic veins to the vertebral venous plexus and subsequently to the brain parenchyma via cerebral venous sinuses [54, 55]. Additionally, secondary spread from pulmonary metastases to the brain is considered a common mechanism underlying brain involvement in CC patients [56, 57, 58, 59].

Our analysis revealed that NEC of the cervix was associated with the highest incidence of BMs, estimated at 10.60 cases per 100 patients, markedly higher than other histological subtypes. A retrospective study by Rangel et al. (2010–2017), using data from Brazil's National Cancer Institute (INCA), reported BMs in 5 out of 25 patients (20%) with cervical NEC. Furthermore, multivariate Cox regression in that study demonstrated a 21.31‐fold higher risk of BMs in NEC compared to SCC [16]. Similarly, an analysis by Park et al. (2010–2019), based on SEER data, found BMs in 11 of 211 patients (5.2%) with NEC. That study also reported a statistically significant difference in BMs rates among patients with NEC versus those with SCC or ADC [14]. The disproportionately higher incidence of BMs in NEC may be attributed to the tumor's unique biological behavior. NECs are aggressive, poorly differentiated malignancies with a typically poor prognosis. Their histopathological identification is often challenging, which can result in delayed diagnosis and presentation at more advanced stages [60, 61]. The combination of late detection and highly invasive potential increases the likelihood of hematogenous spread to distant organs, including the lungs, liver, bones, and ultimately, the brain [56, 62].

Our findings indicated that the mean overall survival following the diagnosis of BMs in patients with CC was 6.80 months. To provide a broader context for survival outcomes in metastatic CC, Yin et al., evaluating 99 CC patients with distant metastases, reported a median overall survival of 11.7 months from diagnosis, with 1‐, 2‐, and 5‐year OS rates of 48.2%, 22.8%, and 12.6%, respectively [63]. Although this study encompassed all metastatic sites and not specifically brain involvement, it provides a broader context for survival outcomes in metastatic CC. In contrast, Wu et al., analyzing SEER data from 2010 to 2019, focused specifically on patients with brain metastases and found a median survival of 6 months, which was considerably shorter than survival among patients with metastases to the lungs (9 months), liver (8.5 months), or bones (11 months) [50]. Collectively, the available evidence suggests that BMs in CC are associated with an extremely poor prognosis. Also, it is important to interpret these survival comparisons in light of the variability in study design, patient selection, and metastatic burden, which contribute to the observed heterogeneity. While variability in reported survival durations across studies may partially reflect lead‐time bias, this does not diminish the clinical severity or prognostic gravity of brain involvement. Early brain imaging may assist in the timely detection and intervention in selected patients [64]. Patients presenting with focal neurologic deficits are more likely to undergo prompt neuroimaging, leading to earlier diagnosis of BMs. However, non‐specific symptoms, such as headache, the most common manifestation, may be misattributed to side effects of chemotherapy or other treatment‐related complications, particularly in patients with mild presentations. This diagnostic ambiguity may result in delayed or missed detection, preventing affected patients from receiving the potential (albeit limited) benefits of earlier intervention [57, 65]. Although BMs are often regarded as incurable, several observational studies have demonstrated that appropriate treatment can significantly prolong median survival. It is important to acknowledge the potential for selection bias in these studies; yet, even under optimal therapeutic conditions, overall survival remains limited following the onset of brain involvement. For patients with multiple BMs, whole‐brain palliative radiotherapy is generally considered the standard approach. In contrast, for patients with isolated brain lesions and no evidence of widespread systemic disease, surgical resection via craniotomy followed by adjuvant radiotherapy may offer improved outcomes. In cases involving disseminated disease, including multiple brain lesions and extracranial metastases, systemic chemotherapy may also be considered as part of a multimodal treatment strategy [64].

While this systematic review and meta‐analysis provide a comprehensive overview of the incidence of BMs in CC patients, along with associated clinicopathological characteristics and survival outcomes, it is important to consider several limitations when interpreting these findings. First, the majority of included studies originated from countries with a high HDI. Despite the higher overall burden of CC in low‐HDI regions, particularly sub‐Saharan Africa, we were unable to identify any studies from these areas that reported on BMs in this population. This lack of representation may have introduced bias in our pooled estimates and limits the generalizability of our findings to lower‐resource settings. Second, there was substantial heterogeneity across the included studies (*I* ^2^ = 94.49%), reflecting differences in study design, patient demographics, disease staging, geographic distribution, and diagnostic approaches. This high variability may have affected the consistency and precision of pooled results, and it is important to interpret our findings in light of this heterogeneity. To address this, we conducted subgroup analyses and meta‐regression to explore potential sources of variability, including temporal trends, study quality, and patient characteristics. Despite these efforts, residual heterogeneity remains, underscoring that the pooled estimates should be considered approximations rather than definitive measures. This variability may have influenced the consistency and precision of pooled results. Finally, a substantial proportion of eligible studies lacked sufficient detail to contribute to the clinicopathological and survival analyses, which led to their exclusion from certain parts of our synthesis. This limitation may have restricted our ability to draw more nuanced conclusions regarding prognostic factors.

## 5 Conclusion

BMs remain a rare but significant complication in CC, strongly associated with poor prognosis and limited survival. This meta‐analysis estimates a global pooled incidence of nearly 1% for BMs in CC patients, with significant regional variations. Although BMs commonly coexist with metastases to other organs, their overall incidence remains relatively low. Our findings highlight that patients with NEC have a significantly higher risk of developing BMs, underscoring the need for targeted evaluation of this subgroup. These insights provide a foundation for future research focused on improving early detection, risk stratification, and tailored therapeutic strategies for this vulnerable population.

## Author Contributions

K.P. and P.E. initiated the research idea and developed the study framework. Literature review and data collection were jointly carried out by K.P., P.E., and A.E.S. Data analysis was handled by P.E. in close collaboration with H‐.A.N., ensuring accurate interpretation of the findings. The manuscript was written and shaped through collaborative drafting by K.P., A.A.‐N., A‐.H.L., A.E.S., P.E., and S.S. M.Y., K.P. and P.E. contributed to the refinement of the manuscript by providing critical insights and revisions. All authors reviewed the final draft, approved the manuscript for submission, and agreed to be accountable for its content.

## Funding

The authors have nothing to report.

## Conflicts of Interest

The authors declare no conflicts of interest.

## Supporting information


**Figure S1:** Sensitivity Analysis of the Studies Included in the Meta‐Analysis, Excluding One Study, for the pooled incidence of brain metastasis from cervical cancer patients.

**Figure S2:** Funnel plot for the publication bias assessment of the studies in meta‐analysis.

**Table S1:** Search strategy.

**Table S2:** Brain metastases cervical cancer information of included studies.

## Data Availability

The data that support the findings of this study are available from the corresponding author upon reasonable request.
