# Supplementary figures and images for: Brain Metastases in Cervical Cancer: A Global Systematic Review and Meta‐Analysis of Incidence and Clinicopathological Features
Source: Cancer Rep (Hoboken). 2025 Nov 26;8(12):e70405. doi: 10.1002/cnr2.70405 (PMC12657064; doi:10.1002/cnr2.70405)

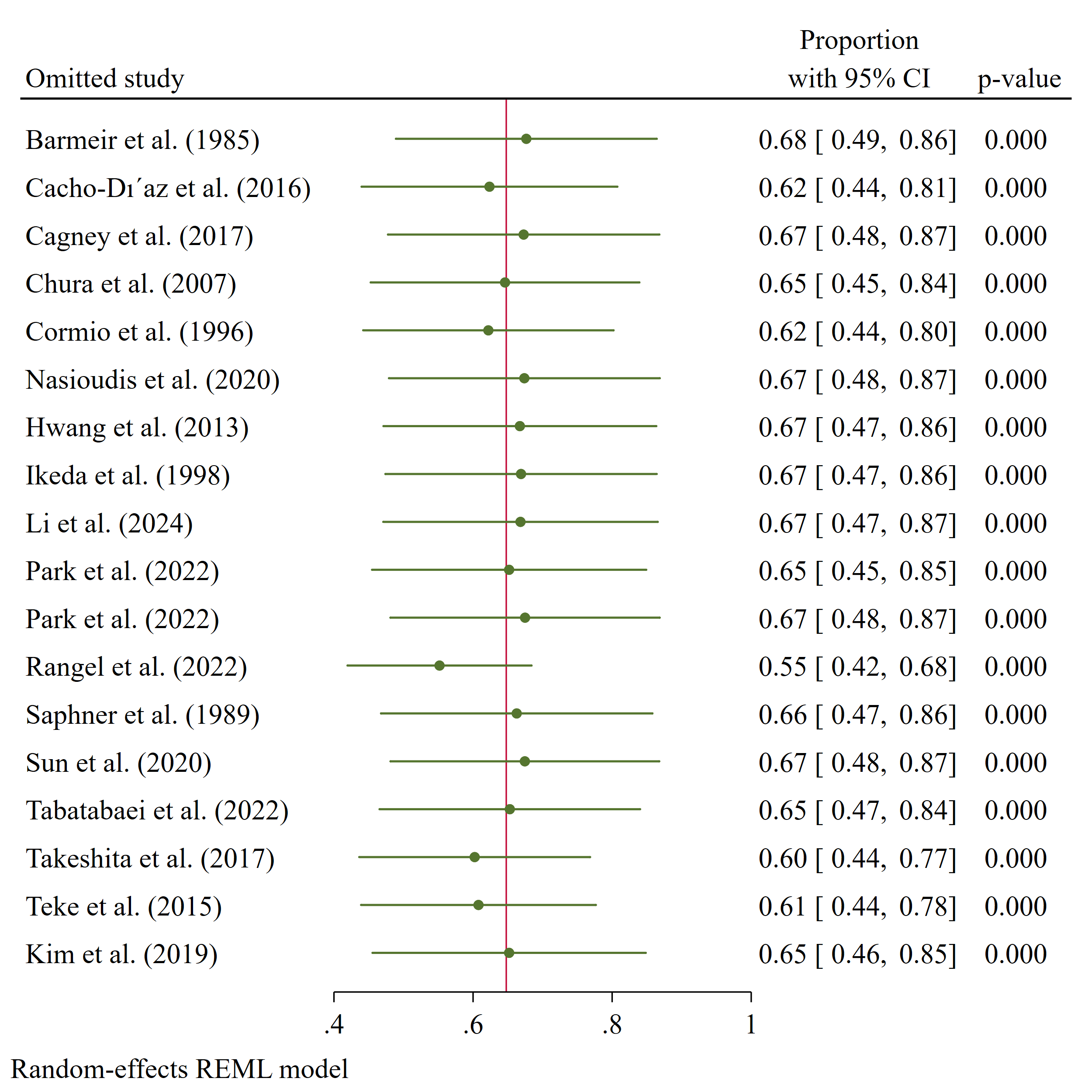

Supplement: Supplementary file 1 — Figure S1: Sensitivity Analysis of the Studies Included in the Meta‐Analysis, Excluding One Study, for the pooled incidence of brain metastasis from cervical cancer patients. [file CNR2-8-e70405-s002.png]

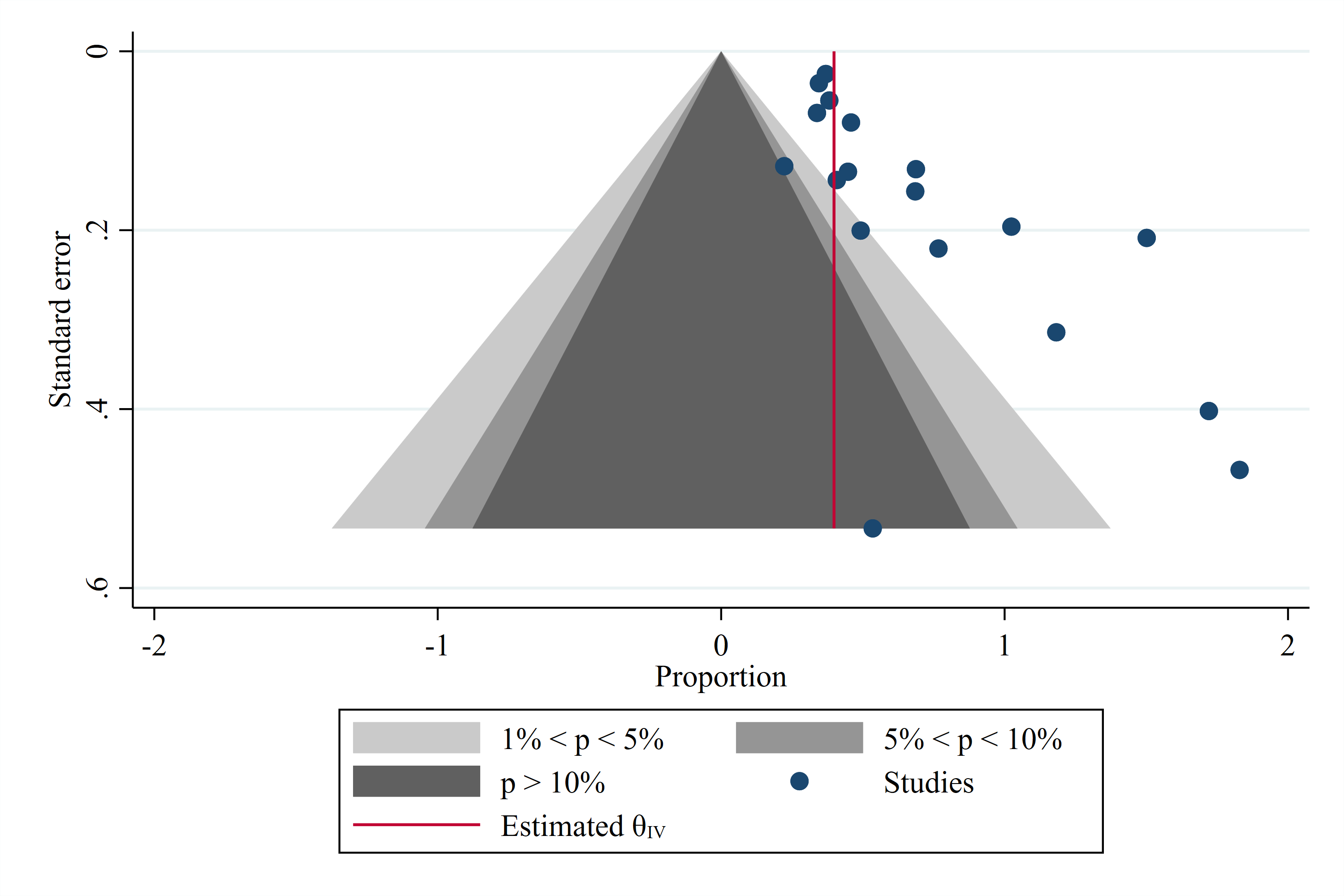

Supplement: Supplementary file 2 — Figure S2: Funnel plot for the publication bias assessment of the studies in meta‐analysis. [file CNR2-8-e70405-s004.png]
