# Supplementary material for: Brain Metastases in Cervical Cancer: A Global Systematic Review and Meta‐Analysis of Incidence and Clinicopathological Features
Source: Cancer Rep (Hoboken). 2025 Nov 26;8(12):e70405. doi: 10.1002/cnr2.70405 (PMC12657064; doi:10.1002/cnr2.70405)
Supplement: Supplementary file 3 — Table S1: Search strategy. [file CNR2-8-e70405-s001.docx]

**Table S1:** Search strategy

| **Source** | **Search strategy** | **Result** |
| --- | --- | --- |
| PubMed (ALL Fields) | (((("Prevalence"[Mesh] OR "Epidemiology"[Mesh] OR "epidemiology" [Subheading]) OR "Incidence"[Mesh]) AND (((("Brain"[Mesh]) OR "Brain Neoplasms"[Mesh]) OR "Cerebrum"[Mesh]) OR (cerebral))) AND (("Neoplasm Metastasis"[Mesh]) OR (metastatic))) AND ("Uterine Cervical Neoplasms"[Mesh]) | 33 |
| Scopus (TITLE-ABS-KEY) | TITLE-ABS-KEY ( ( "Prevalence" OR "Incidence" OR "Epidemiology" OR "Prevalences" OR "Epidemiologies" OR "Epidemics" OR "Morbidity" OR "Outbreaks" OR "Surveillance" OR "Endemics" OR "Occurrence" OR "Frequency" OR "Incidences" OR "Attack Rate" OR "Attack Rates" OR "Rate, Attack" OR "Person-time Rate" OR "Person time Rate" OR "Person-time Rates" OR "Rate, Person-time" OR "Rate, Secondary Attack" ) AND ( "Brain Neoplasms" OR " Brain Neoplasm" OR "Brain" OR "Intracranial" OR "Cerebral" OR "Cerebrum" OR "Cerebra" OR "Encephalon" ) AND ( "Neoplasm Metastasis" OR "Metastase" OR "Metastases" OR "Metastasis" OR "metastatic" ) AND ( "Uterine Cervical Neoplasms" OR "Cervical" OR "Cervix" ) ) | 566 |
| Web of science (Topic) | TS=(("Prevalence" OR "Incidence" OR "Epidemiology" OR "Prevalences" OR "Epidemiologies" OR "Epidemics" OR "Morbidity" OR "Outbreaks" OR "Surveillance" OR "Endemics" OR "Occurrence" OR "Frequency" OR "Incidences" OR "Attack Rate" OR "Attack Rates" OR "Rate, Attack" OR "Person-time Rate" OR "Person time Rate" OR "Person-time Rates" OR "Rate, Person-time" OR "Rate, Secondary Attack") AND ("Brain Neoplasms" OR " Brain Neoplasm" OR "Brain" OR "Intracranial" OR "Cerebral" OR "Cerebrum" OR "Cerebra" OR "Encephalon") AND ("Neoplasm Metastasis" OR "Metastase" OR "Metastases" OR "Metastasis" OR "metastatic") AND ("Uterine Cervical Neoplasms" OR "Cervical" OR "Cervix")) | 139 |
| Embase (ti, ab, kw) | ('prevalence':ab,ti,kw OR 'incidence':ab,ti,kw OR 'epidemiology':ab,ti,kw OR 'prevalences':ab,ti,kw OR 'epidemiologies':ab,ti,kw OR 'epidemics':ab,ti,kw OR 'morbidity':ab,ti,kw OR 'outbreaks':ab,ti,kw OR 'surveillance':ab,ti,kw OR 'endemics':ab,ti,kw OR 'occurrence':ab,ti,kw OR 'frequency':ab,ti,kw OR 'incidences':ab,ti,kw OR 'attack rate':ab,ti,kw OR 'attack rates':ab,ti,kw OR 'rate, attack':ab,ti,kw OR 'person-time rate':ab,ti,kw OR 'person time rate':ab,ti,kw OR 'person-time rates':ab,ti,kw OR 'rate, person-time':ab,ti,kw OR 'rate, secondary attack':ab,ti,kw) AND ('brain neoplasms':ab,ti,kw OR 'brain neoplasm':ab,ti,kw OR 'brain':ab,ti,kw OR 'intracranial':ab,ti,kw OR 'cerebral':ab,ti,kw OR 'cerebrum':ab,ti,kw OR 'cerebra':ab,ti,kw OR 'encephalon':ab,ti,kw) AND ('neoplasm metastasis':ab,ti,kw OR 'metastase':ab,ti,kw OR 'metastases':ab,ti,kw OR 'metastasis':ab,ti,kw OR 'metastatic':ab,ti,kw) AND ('uterine cervical neoplasms':ab,ti,kw OR 'cervical':ab,ti,kw OR 'cervix':ab,ti,kw) | 366 |
