# Supplementary material for: Brain Metastases in Cervical Cancer: A Global Systematic Review and Meta‐Analysis of Incidence and Clinicopathological Features
Source: Cancer Rep (Hoboken). 2025 Nov 26;8(12):e70405. doi: 10.1002/cnr2.70405 (PMC12657064; doi:10.1002/cnr2.70405)
Supplement: Supplementary file 4 — Table S2: Brain metastases cervical cancer information of included studies. [file CNR2-8-e70405-s003.docx]

Table S3- Brain metastases cervical cancer Information of Included Studies

| **ID** | **Author (publication year)^*^** | **Number of Brain metastasis** | **Single Metastasis (Brain)** | **Multiple Metastasis (Brain and other organs (total))** | **Multiple Metastasis (Brain and Bones)** | **Multiple Metastasis (Brain and Lungs)** | **Multiple Metastasis (Brain and Liver)** | **Stage I of cervical cancer** | **Stage II of cervical cancer** | **Stage III of cervical cancer** | **Stage IV of cervical cancer** | **Brain Metastases** | | **Symptoms** | | | | | |
| --- | --- | --- | --- | --- | --- | --- | --- | --- | --- | --- | --- | --- | --- | --- | --- | --- | --- | --- | --- |
|  |  |  |  |  |  |  |  |  |  |  |  | **Single Site** | **Multiple Site** | **Headache** | **Weakness** | **Nausea and Vomiting** | **Paralysis** | **Seizures** | **Confusion** |
| 1 | Barmeir et al. (1985) | 3 | 3 | 0 | NA | NA | NA | NA | NA | NA | NA | NA | NA | NA | NA | NA | NA | NA | NA |
| 2 | Cacho-Dı´az et al. (2016) | 27 | 11 | 16 | NA | 16 | NA | 5 | 17 | 1 | 3 | 9 | 14 | 15 | 5 | 4 | 3 | 1 | NA |
| 3 | Cagney et al. (2017) | 48 | 14 | 34 | 7 | 14 | 2 | NA | NA | NA | NA | NA | NA | NA | NA | NA | NA | NA | NA |
| 4 | Chura et al. (2007) | 12 | 1 | 11 | NA | NA | NA | 6 | 3 | 2 | 1 | 4 | 6 | 6 | NA | 3 | 2 | 2 | 3 |
| 5 | Cormio et al. (1996) | 14 | 7 | 7 | NA | NA | NA | 10 | 3 | 1 | 0 | 6 | 8 | NA | NA | NA | NA | NA | NA |
| 6 | Hwang et al. (2013) | 11 | 0 | 11 | 1 | 8 | 4 | 1 | 2 | 3 | 3 | 2 | 8 | 4 | 5 | 1 | NA | 2 | 1 |
| 7 | Ikeda et al. (1998) | 8 | 0 | 8 | 3 | 2 | NA | 3 | 4 | 1 | NA | 4 | 4 | 3 | 2 | 4 | NA | 2 | NA |
| 8 | Li et al. (2024) | 33 | 16 | 17 | 11 | NA | 4 | NA | NA | NA | NA | NA | NA | NA | NA | NA | NA | NA | NA |
| 9 | Nasioudis et al. (2020) | 211 | 58 | 153 | 72 | 116 | 48 | NA | NA | NA | NA | NA | NA | NA | NA | NA | NA | NA | NA |
| 10 | Rangel et al. (2022) | 51 | 31 | 20 | NA | 12 | 1 | 5 | 13 | 22 | 11 | NA | NA | NA | NA | NA | NA | NA | NA |
| 11 | Saphner et al. (1989) | 6 | 2 | 4 | NA | 4 | NA | NA | NA | NA | NA | 1 | 5 | NA | NA | NA | NA | NA | NA |
| 12 | Sun et al. (2020) | 24 | 4 | 20 | NA | NA | NA | 4 | 3 | 12 | 5 | 8 | 11 | NA | NA | NA | NA | NA | NA |
| 13 | Tabatabaei et al. (2022) | 1 | 1 | 0 | NA | NA | NA | NA | NA | NA | NA | NA | NA | NA | NA | NA | NA | NA | NA |
| 14 | Takeshita et al. (2017) | 18 | 2 | 16 | NA | NA | NA | 2 | 6 | 4 | 6 | 8 | 10 | NA | NA | NA | NA | NA | NA |
| 15 | Teke et al. (2015) | 15 | 5 | 10 | 2 | 5 | NA | 2 | 9 | 1 | 3 | NA | NA | NA | NA | NA | NA | NA | NA |
| 16 | Kim et al. (2019) | 19 | 3 | 16 | NA | NA | NA | 4 | 11 | 0 | 4 | 11 | 8 | 8 | 11 | NA | NA | NA | NA |
| **Total datasets for evaluation** | | **16** | **16** | **16** | **6** | **8** | **5** | **10** | **10** | **10** | **10** | **9** | **9** | **5** | **4** | **4** | **2** | **4** | **2** |
| **Pooled events^*^** | | **501** | **158** | **343** | **96** | **177** | **59** | **42** | **71** | **47** | **36** | **53** | **74** | **36** | **23** | **12** | **5** | **7** | **4** |
| **Pooled Incidence**** | | **-** | **30.76 (19.80 – 41.71)** | **69.24 (58.29 – 80.20)** | **22.97 (12.91 – 33.02)** | **44.10 (30.83 – 57.37)** | **12.27 (2.25 – 22.29)** | **23.26 (11.67 – 34.85)** | **35.53 (23.29 – 47.77)** | **17.94 (7.32 – 28.57)** | **14.83 (8.69 – 20.96)** | **36.44 (28.11 – 44.76)** | **54.56 (46.50 – 62.62)** | **46.58 (35.58 – 57.59)** | **35.80 (16.23 – 55.37)** | **17.11 (7.80 – 26.42)** | **12.45 (2.11 – 22.78)** | **11.02 (0.12 – 21.91)** | **14.50 (0.0 – 29.26)** |

NA; Not Available,

* There is no desired information of brain metastasis in Park et al [1] study.

** The pooled incidence reported in this table is presented based on the number of cases for the variables of interest according to the number of brain metastases, and all values are expressed as percentages.

1. Park HK. Neuroendocrine Carcinomas of the Uterine Cervix, Endometrium, and Ovary Show Higher Tendencies for Bone, Brain, and Liver Organotrophic Metastases. Curr Oncol. 2022;29(10):7461-9. Epub 20221006. doi: 10.3390/curroncol29100587. PubMed PMID: 36290864; PubMed Central PMCID: PMCPMC9600665.
